# Separation of some vitamins in reversed-phase thin-layer chromatography and pressurized planar electrochromatography with eluent containing surfactant

**DOI:** 10.1038/s41598-021-01323-1

**Published:** 2021-11-08

**Authors:** Beata Polak, Emilia Pajurek

**Affiliations:** Department of Physical Chemistry, Medical University of Lublin, Chodźki 4a, 20-093 Lublin, Poland

**Keywords:** Biochemistry, Health care, Chemistry

## Abstract

The separation of some water- and fat-soluble vitamins via micellar systems of reversed-phase high-performance thin-layer chromatography (HPTLC) and pressurized planar electrochromatography (PPEC) was subjected to research. Hence, the influence of the mobile phase composition (surfactant and acetonitrile concentration, eluent buffer pH) on the migration distances and zone separation of some vitamins (thiamine, riboflavin, niacin, pyridoxine, cyanocobalamin, folic acid, ergocalciferol and α-tocopherol) was investigated. Our results indicated that the applied technique has an impact on the solute order. Comparing the system capacity of HPLC and PPEC (measured as height of the theoretical plate) for the mobile phase systems with and without surfactant shows differences, especially for fat-soluble vitamin. The variances and reproducibilities (% RDS) values of the vitamin are less in PPEC than in TLC. Moreover, the migration distances of water-soluble vitamins are longer than fat-soluble ones. Overall, eluent consisting of 50% acetonitrile, 18.75 mM SDS, the buffer of pH 6.99 via the PPEC technique was most appropriate for determining the investigated vitamins in the artificial mixture and the two commercially available vitamin combinations.

## Introduction

Vitamins are essential components of many biochemical processes. As they have regulatory functions (as substrates for the coenzymes synthesis), deficiency or lack of a specific vitamin in the body results in characteristic symptoms, e.g., Beri-Beri disease (Vit. B1), Pellagra (Vit. B3), night blindness (Vit. A) or osteomalacia (Vit. D). Since they are exogenous for humans, their supplementation is necessary. Vitamins are produced primarily by plants and ‘good’ bacteria; however, chemical synthesis is also possible. The amount needed for the proper activity of the body is minimal.

Current literature^1^ broadly presents the influence of vitamin supplementation on human health. In the study of Iwakawa et al., researchers associate dietary intake and blood water-soluble vitamin level (vitamin B2, vitamin C, niacin, and folate) in patients with ulcerative colitis^2^. Chen and co-workers note the correlation of the level of fat-soluble vitamin in the serum of pregnant women or children in Northeast China with the age and the daily regional habits^3,4^.

Vitamin content is sometimes the primary goal of the various sample investigations. This work includes optimising separation conditions before determining vitamins in artificial, natural, or commercially available samples. Moreover, some papers present a determination of water-soluble vitamins in natural products, e.g., rice beans^5^ and wild-growing plants^6^. In such endeavours, distinguishing of water-soluble and fat-soluble vitamins is performed separately. Considering the water-soluble vitamins of the first group, reversed-phase systems of high-performance liquid chromatography are the method of the first choice. For example, the UPLC technique linked with MS/MS detection allowed identifying water-soluble vitamins in paediatric syrup^7^. Furthermore, a comparison of the application of various stationary phases HPLC to separation four fat-soluble vitamins was presented in^8^. In addition, a mixture of fat-soluble vitamins and antioxidants in biological fluids during the single-step process of RP-HPLC was separated by Lazzarino and co-workers^9^.

Some authors have applied thin-layer chromatography to vitamin research. Bito and co-workers employed normal phase thin-layer chromatography to investigate the B12 vitamins in foods^10^. The same method was used to the unsuccessful examination of corrinoid compounds from the fish sauce as a potential source of B12 vitamins in^11^.

The separation of the vitamin mixture is improved by adding a surfactant to a mobile phase. Thus, HPLC with hexane sulphonic acid salt-acetonitrile mixture as the eluent was exploited to investigate B group vitamin in ethnic selected plants from Bangladesh^12^. Some manuscripts present the application of eluent containing a surfactant above critical micelle concentration to the separation of some water-soluble vitamins in HPLC systems^13–15^. After the optimisation of the investigation condition, such eluents were used for the determination of vitamin in different pharmaceutical preparations^13^ or artificial vitamin mixtures^15^.

Micellar mobile phase systems were applied to the separation of fat-soluble vitamins A and E^16^. The micellar HPLC with cloud point extraction was chosen to separate both groups of vitamins in^17^. A review on the application of HPLC micellar systems in water-soluble vitamin research was presented in^18^. A fat-soluble vitamin separation supported by a genetic algorithm in microemulsion liquid chromatography was described by Momenbeik et al.^19^.

The vitamins were also investigated with micellar systems of electromigrational techniques^20,21^. The validated methods were applied to the separation of vitamins in natural samples (food, energy drinks or pharmaceutical preparations) in^20^, or energetic and soft drinks^21^, and multivitamin pharmaceutical formulation^22^.

The pseudo stationary phase formed from the poly (stearyl methacrylate-co-methacrylic acid) micelle was harnessed to investigate and create a system that separated components from commercially available vitamin samples^23^.

The thin-layer chromatography micellar systems were broadly examined by Sumina and co-workers^24–27^. The most valuable facts on sorbent modifications and mechanisms of separation with the use of surfactant-containing eluent are presented in^24^.

The synergistic effect of two surfactants, SDS in stationary phase and Tween 80 as the component of the mobile phase TLC systems, was examined by Mohammad and Zehra to separate water-soluble vitamins^28^. The same authors distinguished cyanocobalamin, thiamine, and ascorbic acid using TLC systems with Tween 80-impregnated silica layers and water as the mobile phase^29^.

Vitamin mixture separation with various techniques and eluent compositions has been the focus of multiple studies. Review on separation with TLC technique was prepared by Pyka (lipophilic vitamin)^30^ and Watanabe and Miyamoto (hydrophilic vitamin)^31^. Moreover, Gentili^32^ and Fanali^33^ published evaluations of the separation of the various vitamin by liquid chromatography. Regarding capillary electrophoresis, a broad review on vitamin separation utilising this approach was presented by Schepdael et al.^34^.

Except the liquid chromatography and electromigrational techniques surfactants (tetrabuthylammonium chloride; betaine, choline chloride) were successfully applied in liquid–liquid microextraction systems as component of deep eutectic solvents. This mode was linked with gas chromatography or spectrophotometry to determine various compounds in edible oil or fruit samples^35–37^.

Our department applied a new hyphen technique to distinguish various mixtures, pressurised planar electrochromatography. It joins two systems of separations—chromatographic (partition) with electromigrational (electrophoretic effect). Compared to TLC, it characterises different selectivity and higher efficiency of separation process^38–40^.

Our study applied micellar systems of high-performance thin-layer chromatography (HPTLC) and pressurized planar electrochromatography (PPEC) to separate water-soluble and fat-soluble vitamins simultaneously. To our knowledge, such experiments in the latter technique were performed and presented for the first time. What is more, research on vitamin separation via TLC seems not to have been undertaken. Our study carried out a comparison of the fat-(hydrophobic) and water-soluble (hydrophilic) vitamin separation using HPTLC and PPEC techniques. The effects of surfactant concentration, the mobile phase buffer pH on the solute retentions and migration distances were considered.

Our work also compared the chromatographic parameters such as peak asymmetry and tailing factors, separation capacity of both techniques (HPTLC and PPEC) with mobile phases with or without surfactant. We then separated artificial and commercially available mixtures of vitamins using both methods. Determination of medical preparations and dietary supplement components using PPEC was presented for the first time.

## Results and discussion

### Effect of surfactant concentration

**Figure 1:**
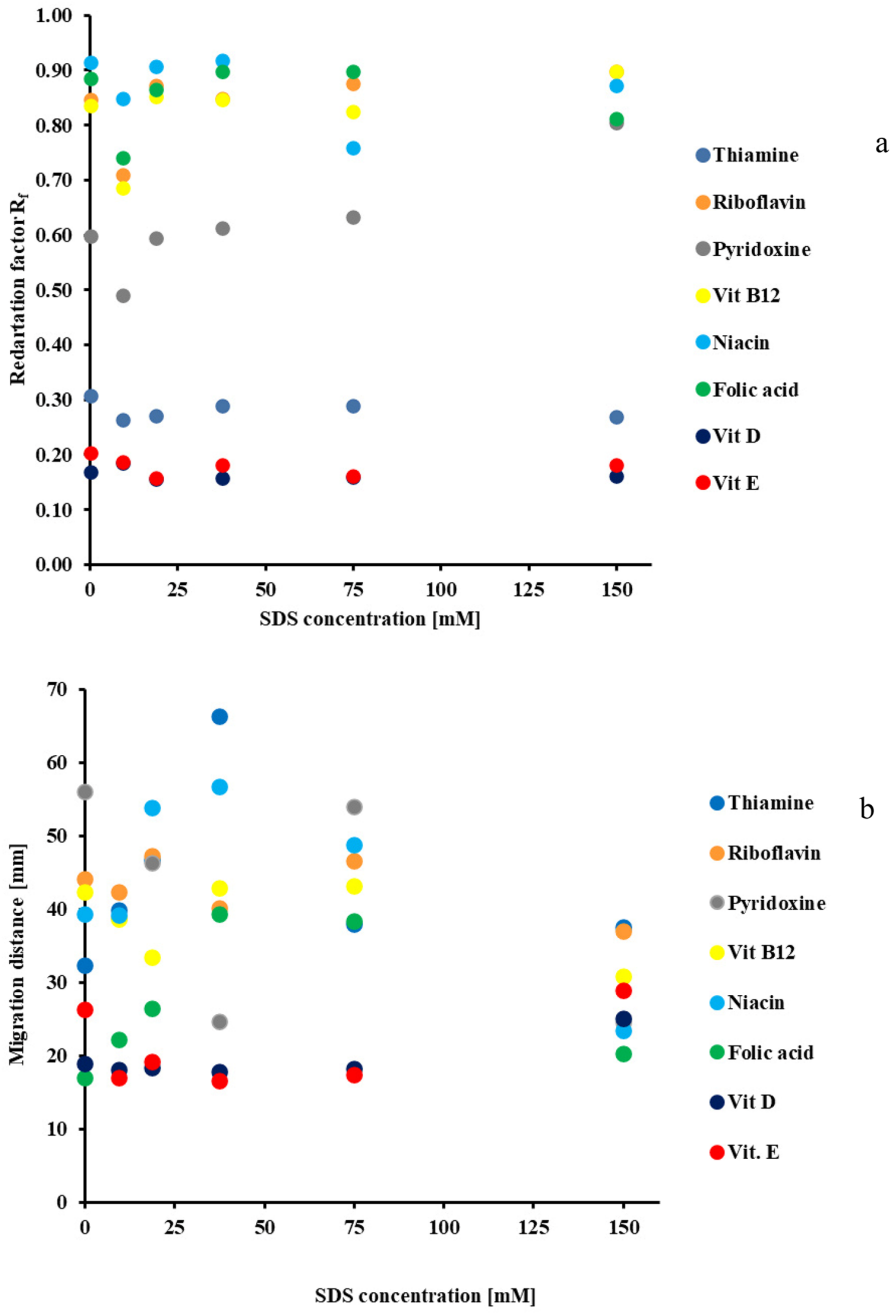
The effect of SDS concentration on investigated vitamins: (**a**)—retention (in TLC) and (**b**)—migration distance (in PPEC). The mobile phase composition was acetonitrile 50% *v*/*v* and aqueous buffer (pH 6.99—composed of acetic acid, phosphoric acid, and boric acid (2.74 mM each), as well as sodium hydroxide (8.60 mM)). For TLC, the distant migration of the eluent front was 45 mm at an ambient temperature of 25 °C. For the PPEC experiment, the time 8 was min., polarisation voltage was 800 V, the temperature was 23 °C.

The effect of the surfactant (sodium dodecylsulphate) concentration on the vitamin retentions and migration distances was presented in Fig. 1a,b.

Since changes in surfactant concentration affect the migration distances of the tested vitamins differently in TLC (see Fig. 1a), we divided the tested compounds into two groups. The first, containing thiamine, vitamins D and E, characterises strong retention, and the concentration of surfactant in the mobile phase has a slight influence upon them. Two of the vitamins mentioned above are fat-soluble and hydrophobic (see log P in Table 3). It explains their high affinity for the non-polar, hydrophobic stationary phase. In contrast, thiamine has the strongest base character among the investigated. Thus, its strong retention results from incorporating thiamine cation into anionic surfactant micelles and strong adsorption onto the non-polar stationary phase.

The second group, containing riboflavin, pyridoxine, cyanocobalamin, niacin, and folic acid, exhibits low retention. The lipophilicity (log P in Table 3) values for these compounds are lower compared to the group described earlier. Thus, their affinity for the mobile phase is greater. The surfactant content in the mobile phase insubstantially affects their migration distances. However, the retention of the water-soluble vitamins increases after adding the surfactant to the mobile phase. A similar effect was observed by Ruiz-Angel and co-workers considering β-blockers and HPLC^41^. Moreover, the retardation coefficient of those solutes is significantly reduced at the lowest SDS concentration in the eluent (9.375 mM) compared to the systems without SDS or containing 18.75 mM SDS. For the fat-soluble vitamins (E and D) and thiamine, there is an insignificant effect of the SDS concentration on the retention.

The SDS content in the eluent, in the investigated concentration range, does not improve significantly solute zone separation. For each eluent, only one vitamin pair was not distinguished (the fat-soluble vitamins (for 75, 18.75 and 9.375 mM SDS) and the water-soluble B2 and B12 (for 37.5 and 150 mM SDS, respectively)).

The sequence of retention of the bands of the investigated compounds for 18.75 mM SDS content in the eluent is as follows (starting from the substances with the weakest retention): niacin > riboflavin and folic acid > vitamin B12 > pyridoxine > thiamine > vitamin D and vitamin E. Such order for most solutes is the same as theoretically determined pK_A_ value increase as shown in Table 3 (folic acid, vitamin B12 and thiamine are an exception).

The effect of surfactant concentration on the migration distance of the investigated compounds in the PPEC technique was presented in Fig. 1b. The PPEC procedure involves the equilibration of the stationary phase with the mobile phase before the experiment^38,39^. This process results in the lack of the unmixing of the eluent components (solvents demixing) during the separation process and the formation of additional mobile phase front lines. It is significant for a mobile phase containing a non-volatile solute (e.g., surfactant), affecting solvent vapour pressures. In a system without prior equilibration (like TLC), two separate mobile phase front lines appear. The effect is particularly noticeable for the eluents containing high surfactant content. Unfortunately, in PPEC such eluents generate a high current in the apparatus when high polarisation voltage is applied. It results in exceeding the technical ability of the power supply, and the voltage is automatically reduced. Our experiments use a lower polarisation voltage (only 800 V) and elongate the experiment time to avoid above mentioned problems.

As shown in Fig. 1b, the surfactant content in the mobile phase influences the solute retention due to changes in the surface of the sorbent. What is more, the concentration of the amphiphile in the eluent phase affects the migration distance of the investigated compounds. Such effect was previously observed in the TLC technique. Introducing even a small amount of SDS to the mobile phase declines the retention of most of the solutes (riboflavin is an exception). In addition, further increasing the SDS concentration in the range of 9.375–18.75 mM elongates the migration distances of the test vitamins. Of note: some solutes (B1, B6, B12, E) exhibit the weakest interactions with the stationary phase for the latter surfactant concentration.

Increasing the amount of SDS in the mobile phase to 37.5 mM reduces the migration distances of most tested vitamins, except for niacin (Vit. B 3), which reaches the longest migration distance. Extending the SDS content in the eluent to 75 mM enhances the migration distances of thiamine, riboflavin, folic acid, vitamins E and D, and shortens the migration of niacin. The highest SDS concentration in the mobile phase reduces the migration distances of most solutes except niacin and vitamin E.

The influence of the SDS content in the eluent on the vitamin order was also observed in the micellar electrokinetic capillary electrochromatography^21,42^. Similar behaviour of different classes solute was noticed in PPEC as well^43,44^.

The most effective system for the solute band separation was the eluent containing 18.75 mM SDS. It turned out that this concentration corresponds to the critical micelle concentration for SDS (see supplementary material). The order of test migration is as follows (starting from the compound with the longest migration distance): niacin > thiamine > vitamin B12 > riboflavin > pyridoxine > folic acid > vitamin E > vitamin D. The resulting migration order differs from that obtained with the TLC technique. However, it agrees with the increasing pK_A_ value in Table 3 for most solutes (thiamine, vitamin B12, and folic acid are an exception). What is more, the PPEC system approach separates all compound bands. This outcome is due to an electrophoretic effect as one of the two retention mechanisms in PPEC. Thus, in subsequent experiments carried out by applying both techniques we have chosen abovementioned SDS concentration.

### Effect of the mobile phase buffer pH

**Figure 2:**
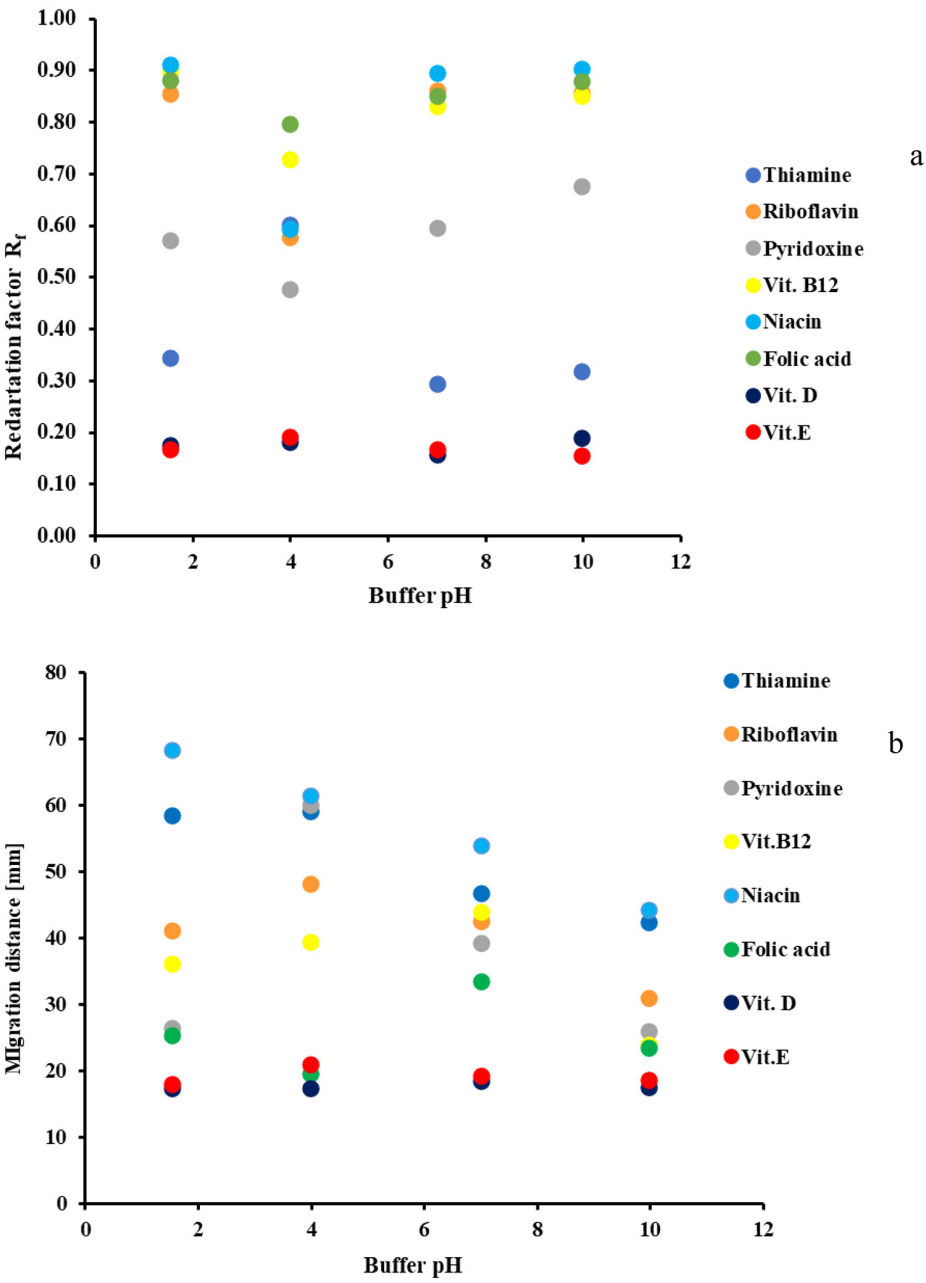
The effect of buffer pH on the investigated vitamins: (**a**) retention (in TLC) and (**b**) migration distance (in PPEC). The mobile phase composition was acetonitrile 50% *v*/*v* aqueous buffer (acetic acid. phosphoric acid and boric acid (4–2.22 mM each) and sodium hydroxide (0–8.86 mM)), as well as 18.75 mM of SDS. For TLC, the distant migration of the eluent front was 45 mm, the temperature was ambient (25 °C). For PPEC, experiment time was 8 min., polarisation voltage was 800 V, temperature was 23 °C.

The effect of mobile phase buffer pH on the solute migration distances was investigated in the next stage of our research. The results are presented in Fig. 2a,b (2a for TLC and 2b for PPEC).

According to physicochemical properties (theoretically determined pK_A_) (see data presented in Table 3), the investigated solutes undergo dissociation processes. On arranging the vitamins according to the diminution of pK_A_ value, the order is as follows: D > thiamine > E > pyridoxine > riboflavin > folic acid > niacin > B12. Due to the high values of pK_A_, the acidic group dissociation of the first three vitamins is very poor, whereas the remaining five undergo the ionisation process to a greater extent. Therefore, investigation of the effect of the mobile phase buffer pH on their retention appears crucial. For the TLC technique, the results of our research in this regard are presented in Fig. 2a. They show that the migration distance of most of the water-soluble vitamins strongly depends on the pH of the mobile phase buffer, while poorly dissociated compound (vitamins D, E, B1) retentions are pH-independent and very strong.

The plot of the ionisable vitamin retention vs eluent buffer pH for TLC forms a V-shape with the minimum at pH 3.99.

For some hydrophilic vitamins (B12, niacin, folic acid, and riboflavin, see Tables 3, 4), a buffer of low pH value (1.54) engenders the weakest retention. Thus, these compounds interact with SDS via hydrophobic effect, and the forming complex has a strong affinity for the mobile phase containing water. As a result, they reach the most extended migration distances. Unfortunately, the association toward the eluent system does not improve solute zone separations.

Vitamin B6 is a base of pK_A_ 9.14 (see Table 3) and shows a moderate retention value at buffer pH 1.54.

Increasing the eluent buffer pH to 3.99 enhances both dissociations of the first described group (vitamins B12, B9, B3, B2, B6) and interactions within SDS micelle. This effect is due to the outside of the vitamin-SDS complex having a slightly negative charge, leading to strong retention onto the sorbent surface.

Further increase of the mobile phase buffer pH to 6.99 and 9.97 enhances the affinity of the solutes toward the eluent due to repulsion effects between silanol groups of the stationary phase and negatively charged micelles.

The best separation of the tested compound bands was observed for the buffer pH of 3.99 and 6.99.

Since the PPEC technique involves the electrophoretic effect during solute migration, and this is pH-dependent, the role of buffer pH in the compound separation process is crucial. Hence, we investigated the impact of the mobile phase buffer pH on the solute migration distances. The results are presented in Fig. 2b. It should be stressed that for the extreme buffer pH values (1.54 and 9.97), the solute migration distances significantly differ from the results obtained for the remaining pH values. For the lowest pH buffer applied (1.54), various solute hydrophobicity and electroosmotic flows cooperate. This buffer pH is below the pK_A_ value for acidic vitamins (niacin, folic acid and B12), so their hydrophobicity is high. However, it is not high enough to cause strong interactions with the stationary phase resulting in shortening migration distances. Thus, at pH 1.54, niacin shows the longest migration distance. Simultaneously, overlapping electrophoretic and electroosmotic effects toward the cathode elongates of B2 and B6 vitamin cation migration.

Regarding the buffer of the highest pH value (9.97), the migration distance of the solutes is the result of the competition between electroosmotic flow rate and interaction with the stationary phase. As for the acidic vitamin (B12, niacin and folic acid) and SDS system, their hydrophobic interactions act against the EOF and shorten migration distances. Strong retention is also observed for vitamins of moderate and high pK_A_ (B1, B2, B6, D and E). Such behaviour results from the increased hydrophobicity of complexes with SDS micelles and strong interaction with the stationary phase.

Considering 3.99 and 6.99 pH values, strong EOF overrides vitamin-SDS micelle complex interactions with the stationary phase. The mobile phase of buffer pH 3.99 reduces the hydrophilic character of the abovementioned complex since vitamins are neutral or slightly charged. The outcome of this effect is the longest solute migration distances for most of the vitamins (B1, B2, B6, and E) due to EOF. Interestingly, folic acid migrates the shortest distance at this buffer pH. It is due to the negative charge of this vitamin at pH close to pK_A2_.

At pH 6.99, the hydrophilic character of complex micelle increases due to vitamin dissociation. The EOF is high enough to elongate the migration distances for anions of vitamin B12 and folic acid.

For some solutes (B2, B12), applying the PPEC technique results in the plot migration distance vs buffer pH shape reversal to TLC. Vitamins B1, B2, B6, B12 exhibit the maxima of the migration at pH 3.99. For pH 6.99, vitamins B9, D and E demonstrate the highest migrations. Vitamin B9 (folic acid) shows an almost straight descending line of migration distance vs mobile phase pH plot. In contrast, in the TLC technique it does not exhibit such behaviour. In PPEC, such a shape is due to the electrophoretic effect.

Our work reveals that the separation of the vitamin zones is pH-dependent. At pH 6.99, the zones are well separated, and only one pair (B1 and B6) reveals solute overlap.

Since the better zone separation was observed at the mobile phase with the buffer of pH 6.99, this system was chosen for further investigations.

### Effect of the acetonitrile concentration

Changes in the concentration of the polar mobile phase modifier affect the retention of the tested substances in each chromatographic process, including micellar chromatography. The results are presented in graphs 3a (for TLC) and 3b (for PPEC).

**Figure 3:**
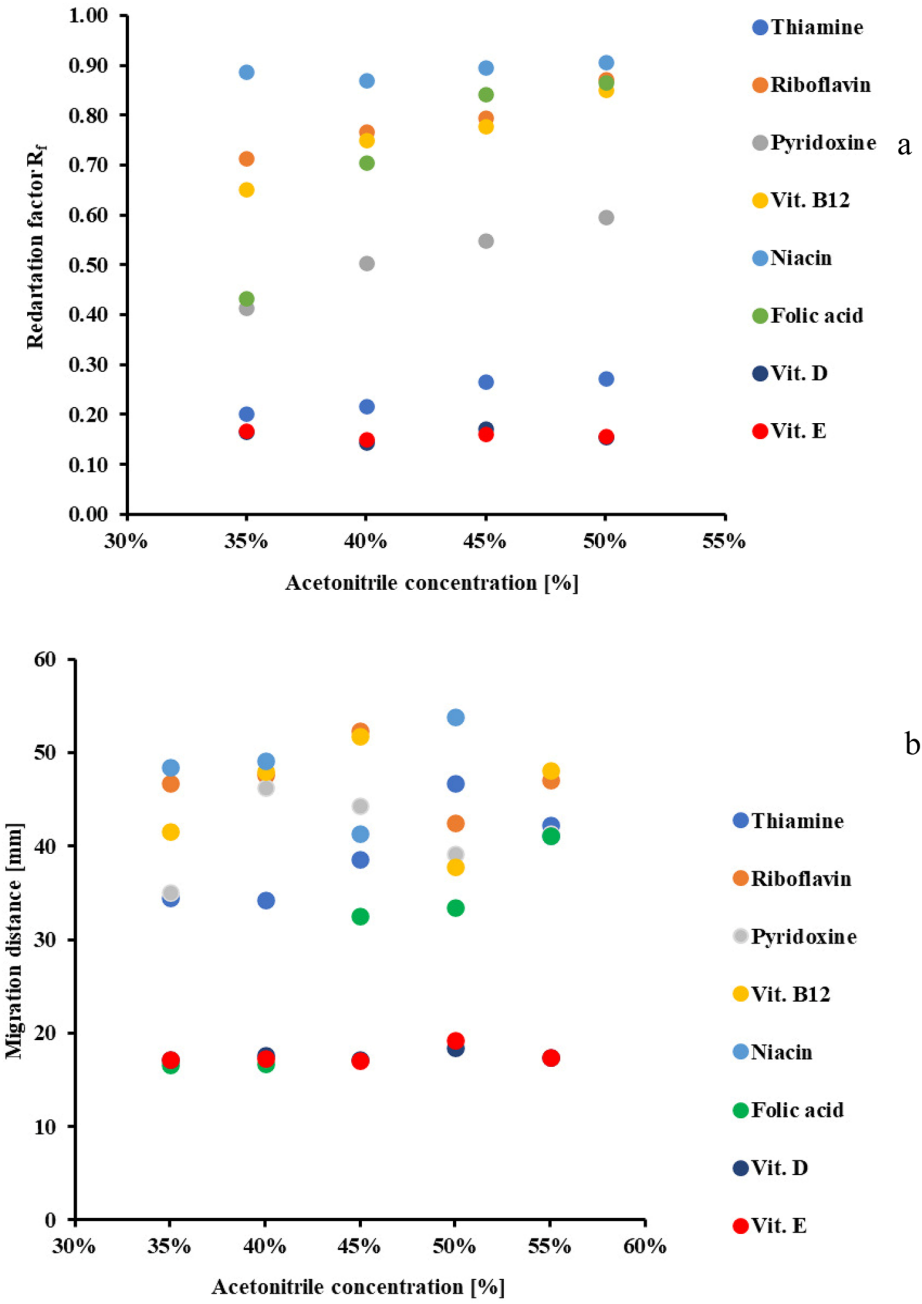
The effect of acetonitrile concentration on the investigated vitamin: (**a**) retention (in TLC) and (**b**) migration distance (in PPEC). The mobile phase composition consisted of aqueous buffer of pH 6.99 (acetic acid. phosphoric acid and boric acid (2.74 mM each) and sodium hydroxide (8.60 mM)), 18.75 mM of SDS and various acetonitrile content. For the PPEC technique, polarisation voltage was 800 V, experiment time was 8 min and the temperature was 23 °C.

Regarding the TLC technique, similarly to previous papers^38,39^, acetonitrile concentration diminishes the retention of the tested vitamins (Fig. 3a). Indeed, the R_f_ coefficients of hydrophobic compounds of high log P values (vitamins D, E and B1 see Tables 3, 4) hardly change with the content of mobile phase modifier being enhanced. Simultaneously, in the same conditions, the retention of the remaining water-soluble vitamins decreases.

The highest values of solute R_f_ were obtained for the highest acetonitrile content (50%) in the mobile phase. Moreover, systems containing a higher concentration of the modifier (40–50%) demonstrate the best solute band separation. The acetonitrile content in the mobile phase also affects the solute order. Thus, the retention of riboflavin and folic acid differs for 40 and 45% of acetonitrile. The sequence of migration of the substance bands for the eluent with 50% acetonitrile is as follows (starting with the compound with the weakest retention): niacin > folic acid and riboflavin > B12 > B6 > B1 > D and vitamin E.

The increased acetonitrile content in the mobile phase changes the value of critical micelle concentration (CMC) and affects the formation of micelles. What is more, the molecules of acetonitrile are incorporated into the micelle. Thus, we are dealing with a system with hybrid micelles. Such micelle types were reported earlier in literature^42–44^.

Figure 3b shows the plot of acetonitrile concentration in the mobile phase vs the solute migration distances for the PPEC technique. As in TLC, compound retention depends on the acetonitrile concentration in the mobile phase. The lowest acetonitrile concentration (35%) shortens migration distances, while the concentration of 45 or 50% elongates. Of interest, the highest acetonitrile content (55% *v*/*v*) decreases the solute migration distance. It may be due to disturbances in the micelle structure caused by too high acetonitrile content.

Similarly to TLC, hydrophobic compounds (vitamins D and E) migrate the shortest distances, and the vitamin zones mentioned above separate poorly. Contrary to TLC results, vitamin B1 is characterised by weaker retention. This outcome is the result of the electrophoretic effect.

In our experiments, 50% acetonitrile in the mobile phase resulted in the best separation of the bands of the tested compounds. The order of elution of the solutes for this concentration is (starting with the compound with the longest migration distance): folic acid > niacin > thiamine > pyridoxine > riboflavin > vitamin B12 > vitamin D > vitamin E. In PPEC, the sequence of substance bands differs from TLC due to the electrophoretic effect. As the best results in both techniques were obtained in the concentration range of 45–50%, we used a mobile phase containing 50% acetonitrile in subsequent experiments.

### Comparison of TLC and PPEC techniques

To compare and evaluate TLC and PPEC techniques on the separation capacity and reproducibility, we used two vitamins (B12 and E) as test solutes. These demonstrated different retention. B12 exhibited stronger interactions in the mobile phase, thus migrated longer distances, whereas E is bonded by the sorbent, so its migration distance was short. The obtained results are presented in Tables 1 and 2.

**Table 1:** Statistical evaluation of PPEC and TLC experimental results.

	Vit. B12		Vit. E	
	PPEC	TLC	PPEC	TLC
No	8	8	8	8
Average migration distance [mm]	51.38	36.72	16.02	6.71
Median [mm]	51.45	36.95	16.10	6.70
Variance [mm]	0.12	1.45	0.04	0.27
%RDS of migration distance	0.66	3.00	1.20	7.80

The mobile phase consisted of 50% acetonitrile, 18.75 mM SDS and a buffer solution of pH 6.99 ((acetic acid, phosphoric acid, and boric acid (2.74 mM each) and sodium hydroxide (8.60 mM)). The PPEC polarisation voltage applied was 1200 V, experiment time was 6 min. TLC eluent migration distance was 5 mm, experiment time was 11 min 30 s*.* Solutes of vitamins B 12 and Vit. E were investigated.

**Table 2:** A comparison of the PPEC and TLC experimental results.

	System without SDS				System with SDS (18.75 mM)			
	Vit B12		Vit E		Vit. B12		Vit E	
	TLC	PPEC	TLC	PPEC	TLC	PPEC	TLC	PPEC
Migration distance (mm)	36.65	42.70	9.20	26.40	36.72	51.38	6.71	16.02
Height of the theoretical plate, H_obs_^a^ (μm)	4.00	26.00	287	56.00	6.00	27.20	69.10	30.40
Asymmetry coefficient (A_s_)^b^	0.57	0.71	0.27	0.62	0.98	0.96	1.12	1.07
Tailing coefficient (T_F_)^c^	0.79	0.85	0.64	0.81	0.97	0.91	1.24	1.14

Here, the mobile phase consisted of 50% acetonitrile, 18.75 mM SDS and a buffer of pH 6.99 ((acetic acid, phosphoric acid, and boric acid (2.74 mM each) and sodium hydroxide (8.60 mM)). PPEC polarisation voltage applied was 1200 V, experiment time was 6 min. TLC eluent migration distance was 45 mm, experiment time was 11 min 30 s. Test solutes: Vit. B 12 and Vit. E

Both peak tailing and asymmetry factor formulas were taken from^46^.

^a^The height of the theoretical plate was calculated using the following equation: $H_{obs} = \frac{{\sigma^{2} }}{{Z_{x} }}$, where σ is the half of peak width at 0.607 height and Z_x_ is solute zone migration distance.

^b^Peak asymmetry factor was calculated using the following equation: A_s_ = b/a, where b is the distance from the peak midpoint (perpendicular from the peak highest point) to the trailing edge of the peak measured at 10% of peak height (left peak half-width) and a is the distance from the leading edge of the peak to the peak midpoint (perpendicular from the peak highest point) to the trailing edge of the peak measured at 10% of peak height (right peak half-width).

^c^Peak tailing factor was calculated using the following equation: T_f_ = (a + b)/2a, where b is the distance from the peak midpoint (perpendicular from the peak highest point) to the trailing edge of the peak measured at 10% of peak height (left peak half-width) and a is the distance from the leading edge of the peak to the peak midpoint (perpendicular from the peak highest point) to the trailing edge of the peak measured at 10% of peak height (right peak half-width).

The variances and reproducibilities (% RDS) of the vitamin migration distances are smaller in PPEC in contrast to TLC (see Table 2). What is more, the % RDS for vitamin E result in TLC is very high (7.8%).

A comparison of separation capacity (measured as theoretical plate height (H)) of both techniques in systems with and without surfactant is presented in Table 2. Considering the theoretical plate height (H) of chromatography and Vit B12 as a test solution, the TLC system is very efficient. The H value is low for both systems with and without surfactant. Thus, we noticed that the surfactant has an insignificant effect on the H value with this vitamin. The H value tested with vitamin B12 for the PPEC technique was notably low and did not reach TLC values.

Regarding vitamin E as test solute, the H values of both systems with the surfactant indicate that both systems had gained efficiency. For the PPEC system with SDS, the efficiency coefficient was five times lower than the system without amphiphile. For TLC, the mobile phase with surfactant had a two times lower H than it did without the amphiphile. Thus, surfactant improves the system efficiency of both TLC and PPEC techniques in a system with fat-soluble E vitamin as the test solute. The significantly lower H value for PPEC and vitamin E as test solute compared with TLC confirms the advantage of the previous over the latter system.

Bearing in mind the solute zone shape being depicted as asymmetry (A_s_) and the tailing (T_f_) coefficient, both the presence of surfactant and the technique applied affect the factors mentioned above. We saw those systems without SDS exhibit non-Gaussian peak shapes with zone fronting (A_s_ lower than 1), and additionally, the technique used has an impact on the zone profile. Thus, for PPEC, the A_s_ values for both tested solutes are higher than that for HPTLC. Moreover, we noted that the solute type affects the A_s_ and T_f_. Consequently, for vitamin E, both coefficients are lower in comparison to that of Vit B12. Moreover, a strong zone fronting is observed for TLC (A_s_ = 0.27).

Considering the systems with SDS, the peak zone shapes are closer to Gaussian (A_s_ values are near to 1.0) for both techniques. In addition, there are insubstantial discrepancies between HPTLC and PPEC regarding the solute tested. Furthermore, the A_s_ and T_f_ values for vitamin B12 and TLC are closer to 1.0 when compared with PPEC. In contrast, as mentioned earlier, the coefficient values are comparable to 1.0 for PPEC and vitamin E as test solutes. Thus, the presence of SDS improves the shape of solute zones in both TLC and PPEC.

### Separation of vitamin mixtures

After previous experiments and choosing the best conditions, an attempt was made to separate an artificial vitamin mixture. Since the B3 vitamin exists in many forms, we decided to use both PPEC and TLC to distinguish two B3 vitamins (niacinamide and niacin). For this purpose, the standard band of niacinamide was applied to the sorbent as a separate zone. We found the migration distance of this compound to be shorter in comparison with that of niacin. Such behaviour is known from the literature^21,45^.

Thus, our artificial mixture contains E, B1, B2, B3 (in two forms-niacin and niacinamide), B6 and B12 vitamins.

**Figure 4:**
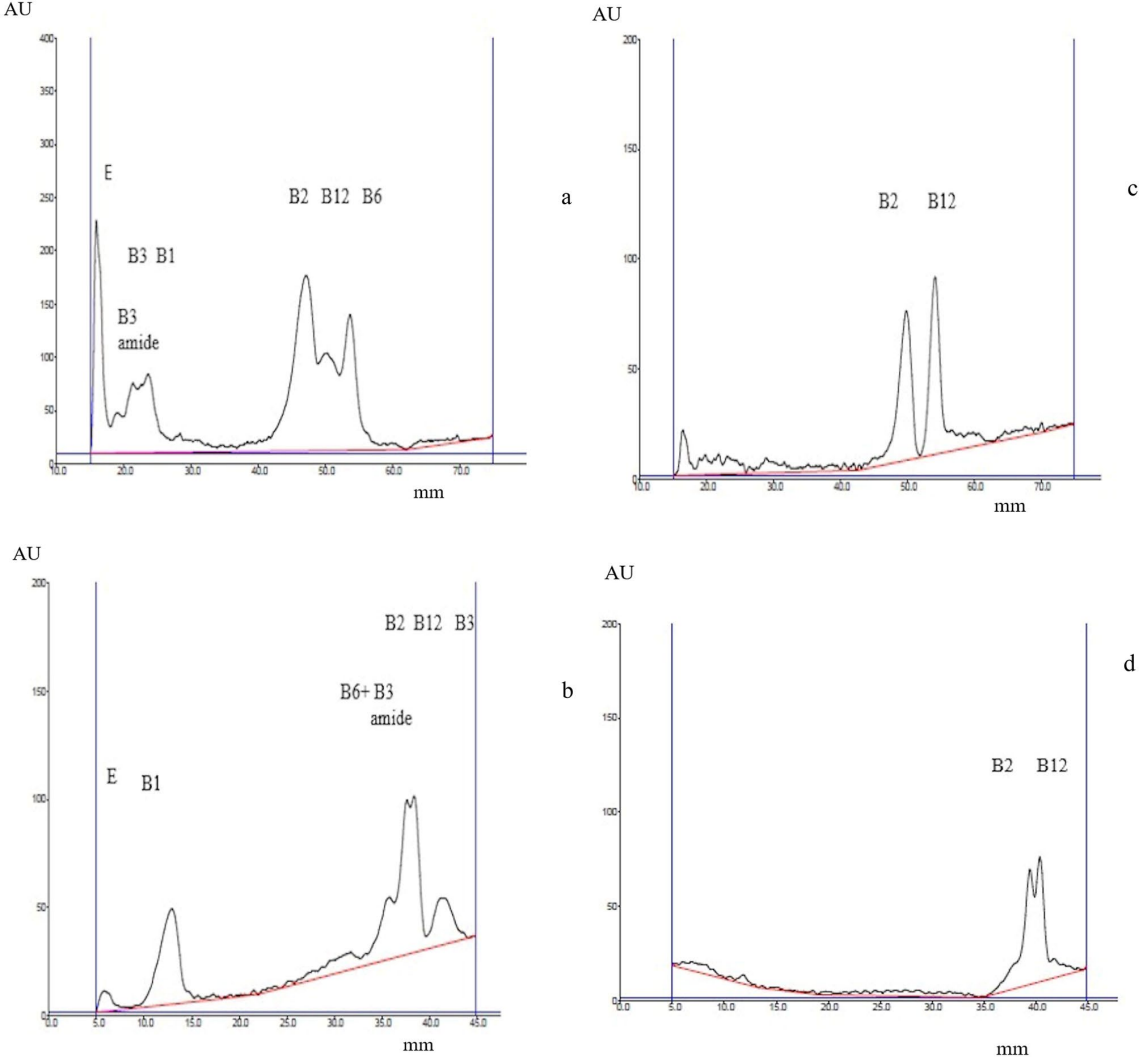
Separation of vitamin mixture compositions by means of PPEC (**a**,**c**) and TLC (**b**,**d**). The mobile phase was 50% acetonitrile, a buffer of pH 6.99 ((acetic acid, phosphoric acid, and boric acid (2.74 mM each) and sodium hydroxide (8.60 mM)), as well as 18.75 mM SDS. UV detection was at 254 nm (**a**,**b**) and 361 nm (**c**,**d**).

Chromatograms and electrochromatograms of this mixture separation are presented in Fig. 4. Since the polarization voltage applied in PPEC technique was higher than in previous experiments, the sequence of the vitamins in mixtures was based on prior separation of standards.

Regarding the mixture component order, there are discrepancies between TLC (Fig. 4a) and PPEC (Fig. 4b). In the latter, both B-3 vitamins migrated shorter distances in comparison to the former. Moreover, the migration distance of B6 varied to the technique used. As for PPEC, the migration distance was longer.

Finally, we decide to ascertain the possibility to separate two commercially available vitamin mixtures (Neurovit and Tiolip) through PPEC and TLC. The first one contains vitamins B1, B6 and B12, as well as supplementary substances. The second consists of vitamins E, B1, B2, B6 and other compounds (pantothenic acid; selenium salt, α-lipoic acid, and γ-linolenic acid).

**Figure 5:**
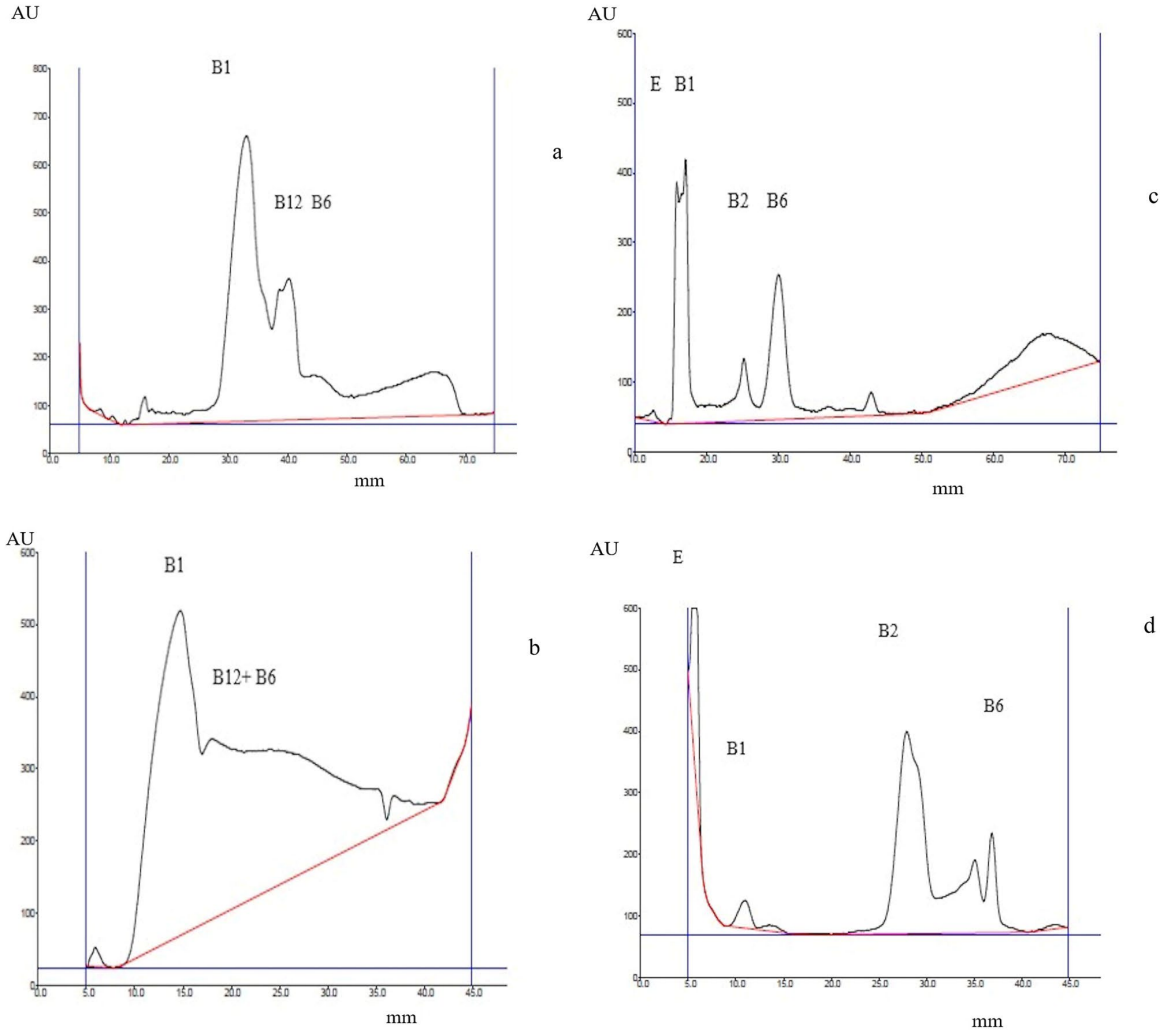
Separation of commercially available vitamin mixtures (Neurovit (**a**,**b**) and Tiolip (**c**,**d**) by way of applying PPEC and TLC techniques. The mobile phase consisted of 50% acetonitrile, a buffer of pH 6.99 ((acetic acid, phosphoric acid, and boric acid (2.74 mM each) and sodium hydroxide (8.60 mM)), as well as 18.75 mM SDS. For Neurovit and the PPEC technique (**a**), the experiment was carried out for 4 min using a polarisation voltage of 1200 V. For Tiolip (**c**), the PPEC technique was applied for 6 min, and the voltage was 1200 V. For both drugs, the TLC (Neurovit (Fig. 5b), Tiolip (**d**) experiments lasted about 11 min. Detection was at 254 nm.

The chromatograms and electrochromatograms presented in Fig. 5 show that it was possible to use PPEC and TLC techniques to separate the vitamin components in Neurovit and Tiolip. As for Neurovit (Fig. 5a,b), the migration distances of the solutes were much longer for the PPEC technique (Fig. 5a) than for TLC (Fig. 5b). Unfortunately, the latter technique does not distinguish all solute peaks. The order of solutes on the chromatogram was found to be the same as the pK_A_ value decrease (see Tables 3, 4).

**Table 3:** Some physicochemical properties of the investigated vitamins (theoretical values of pK_a_ were found in Drug-bank).

Solute	logP	pK_a_	Detection wavelength λ [nm]
Vitamin B1 (thiamine)	− 2.10	15.54	254
Vitamin B2 (riboflavin)	− 1.00	6.97	254 and 361
Vitamin B3 (niacin)	0.29	2.79	254
Vitamin B6 (pyridoxine)	− 0.57	9.40	295
Vitamin B12 (cyanocobalamin)	− 14.00	1.82	361
Vitamin B9 (folic acid)	− 0.68	3.37	295
Vitamin D (ergocalciferol)	7.59	18.38	254
Vitamin E (α-tocopherol)	8.84	10.80	254

**Table 4:**
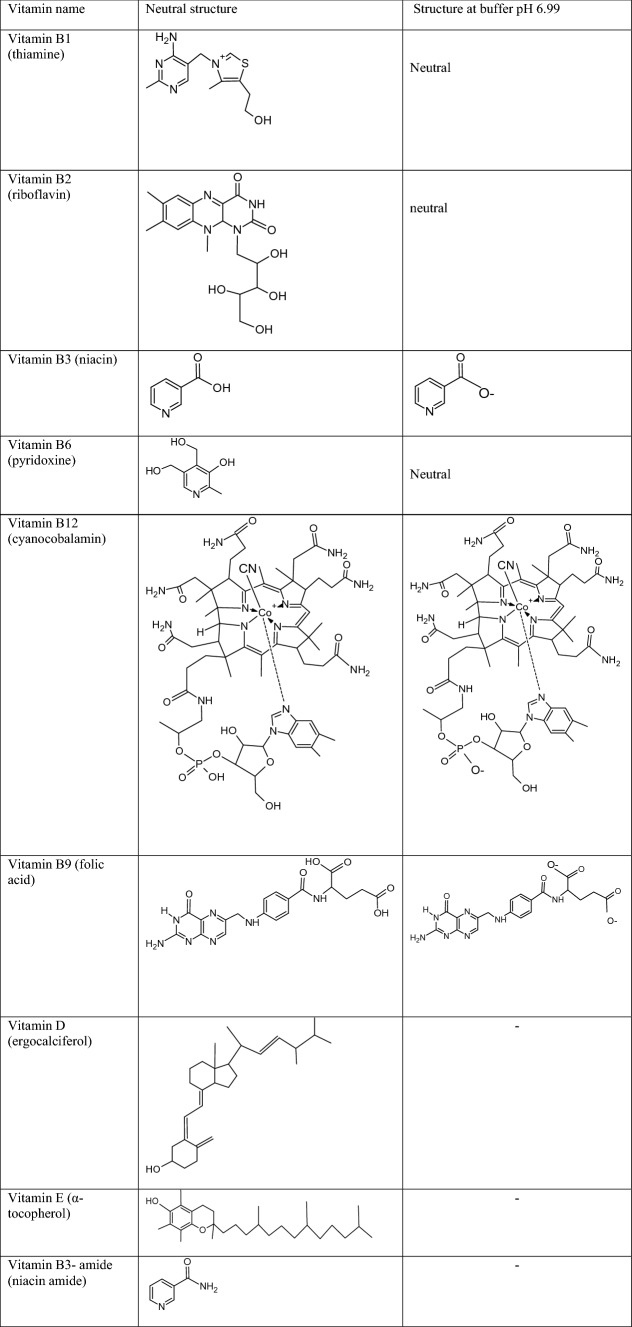
Chemical structures of investigated compounds in neutral and ionic forms at buffer pH 6.99.

Regarding the dietary supplement Tiolop (Fig. 5c,d), which contains various compound including vitamins, the chromatogram (Fig. 5d) and electrochromatogram (Fig. 5c) show that despite the complexity of the formulation, the composition of the mobile phase and experiment conditions allowed effective solute separations. The sequence of the vitamin bands obtained by both techniques was as follows: E, B2, B6 and B1, and the order of identified vitamins on the electrochromatogram was analogous to their hydrophobicity decrease. Both the chromatogram and the electrochromatogram, apart from the peaks of vitamins, also showed other not identified substance bands.

## Conclusions

Mobile phase composition (both surfactant and acetonitrile) and mobile phase buffer pH influenced the vitamin retentions in PPEC and TLC techniques. We saw that sorbent equilibration with the mobile phase before beginning the PPEC experiment improve the solute separation due to the lack of the second eluent front.

Our work indicates that the order of some vitamin-SDS complexes is dependent on the technique applied. In addition, whether using TLC and PPEC, the hydrophobic (fat-soluble) D and E vitamins exhibited strong affinity toward the non-polar stationary phase. The outcome of our work reveals that the reproducibility of the migration distances measured via PPEC application is significantly better than that of TLC usage. Moreover, the presence of surfactant in the eluent enhanced separation capacity for hydrophobic vitamins (vitamin E) as test solutes while having an insignificant effect on hydrophilic vitamins (vitamin B12). Regarding solute peak shape (depicted as A_s_ or T_f_ factors), the presence of surfactant makes it close to the Gaussian profile no matter if PPEC or TLC is applied. Applying the high polarisation voltage in the PPEC technique almost two times shortens the experiment time compared to the time in TLC.

We conclude that, due to its benefits, PPEC can be successfully applied, together with a micellar mobile phase, to separate vitamin mixtures.

## Methods

### PPEC experiments

PPEC experiments were performed with a device composed of PPEC chamber, high voltage power supply (E 752, max. voltage 5 kV, Consort, Belgium), an ammeter and hydraulic press (Współpraca, Lublin, Poland)^38^. The PPEC procedure was performed according to that previously described^39^. The wetting time of the adsorbent layer was 20 s. Teflon foil was pressed onto the adsorbent layer of the chromatographic plate at a pressure of 32 bars.

## Materials

Mobile phase solutions were prepared by mixing acetonitrile (Avantor Performance Materials, S.A. Gliwice, Poland) with a buffer solution containing surfactant. Buffers were prepared by titrating a basic solution (phosphoric acid, acetic acid (both from Avantor Performance Materials Poland S.A., Gliwice, Poland) and boric acid (Standard, Lublin, Poland) each 0.04 M solution) with sodium hydroxide (0.2 M solution). The pH values of the buffer solutions were determined via pH-meter CP-551 (Elmetron, Zabrze, Poland). All mobile phase components used in the investigations were pure grade and supplied by Avantor Performance Materials SA (Gliwice, Poland). The surfactant used in experiments (sodium dodecylsulphate (SDS)) was purchased from Merck (Darmstadt, Germany).

Standards of the following vitamins: thiamine hydrochloride (vitamin B1), niacin (vitamin B3), pyridoxine hydrochloride (vitamin B6), cyanocobalamin (vitamin B12), folic acid, ergocalciferol (vitamin D) and α-tocopherol (vitamin E) were purchased from Sigma-Aldrich (Milwaukee, USA). Riboflavin (Vitamin B2) was isolated from tablets produced by TEVA, Warsaw, Poland. The physicochemical properties of the investigated solutes are presented in Table 1. During experiments, two commercially available vitamin preparations were investigated: Neurovit (GL-Pharma; Lannach, Austria) and Tiolip (Selenium; Mylan; USA). The first drug is a mixture of vitamins B1, B6 and B12. The second consists of alfa lipoic acid, gamma-linolenic acid, vitamins E, B1, B2, B6 and pantothenic acid.

Solutions of water-soluble vitamins were prepared by dissolving 1 mg of the compound in an ethanol–water mixture (53% v/v). In contrast, the fat-soluble vitamins were dissolved in ethanol. The final concentration of vitamins was 0.07% w/v. The solutions were refrigerated. The physicochemical properties of some vitamins are listed in Tables 3, 4. The vitamin spots were detected at 254 nm, 295 nm and 361 nm.

Vitamins were isolated from the Neurovit product after tablet micronisation employing extraction using an ethanol–water mixture and separation of solid particles by filtration. The final vitamin concentration was 0.07% w/v. Since the Tiolip is a fluid in a soft capsule, its interior was dissolved in an ethanol–water (80:20 v/v) mixture and then filtered.

10 cm × 10 cm HPTLC RP-18 W plates (Merck, Darmstadt, Germany) were used in the experiments.

Silicone sealant components Sarsil W, Sarsil H50 and hardener were purchased from Zakłady Chemiczne “Silikony Polskie” Sp. z O.O., Nowa Sarzyna, Poland.

### PPEC plate preparation

The plates were cleaned using methanol by being dipped in this solvent for 1 min. The plates were then air-dried and placed in an oven at 105–110 °C for 15 min and left in a desiccator for cooling. A margin of 4 mm width of silicone sealant was subsequently formed on the whole periphery of each chromatographic plate according to the procedure described in a previously published paper^38^.

### Application of investigated compounds

Solutes were applied to the chromatographic plates using the aerosol applicator ATS 4 (Camag, Muttenz, Switzerland). The applied sample volumes were 4 µL.

### Chromatogram development

HPTLC development was performed in a horizontal DS chamber for TLC (model DS-II-10 × 10, Chromdes, Lublin, Poland). Chromatograms were developed from the prepared chromatographic plates after 15 min equilibration of the chamber atmosphere. The migration distance of the mobile phase was 45 mm.

Chromatogram and electrochromatogram detection were performed via CAMAG TLC Scanner 4 (CAMAG, Muttenz, Switzerland).

The values of migration distance of solutes were the average of triplicate experiments.

### Ethical approval

This article does not contain any studies with human participants or animals performed by any of the authors.

## Supplementary Information

Supplementary Figure S1.
