# Supplementary material for: Separation of some vitamins in reversed-phase thin-layer chromatography and pressurized planar electrochromatography with eluent containing surfactant
Source: Sci Rep. 2021 Nov 8;11:21851. doi: 10.1038/s41598-021-01323-1 (PMC8575784; doi:10.1038/s41598-021-01323-1)
Supplement: Supplementary file 1 — Supplementary Figure S1. [file 41598_2021_1323_MOESM1_ESM.pdf]

Supplementary material

Spectrophotomeric determination of critical micelle concentration for the surfactant in the mobile phase

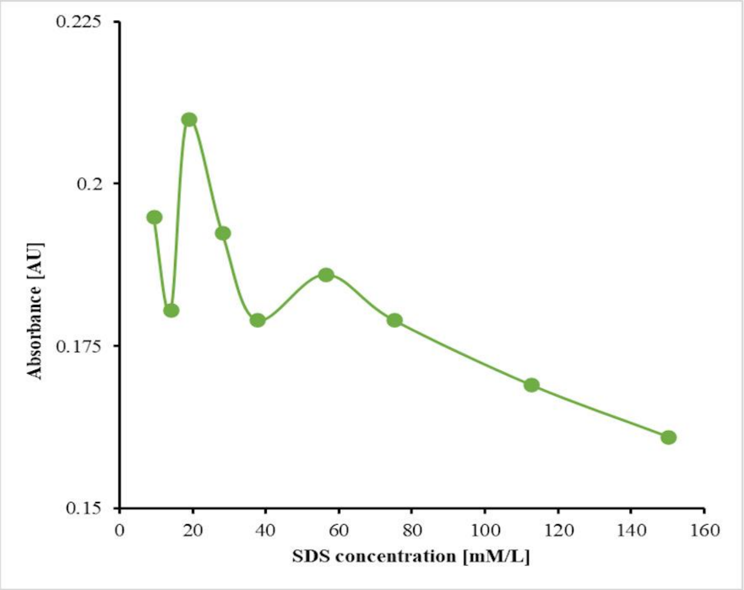

Fig. 1. Determination of critical micelle concentration of the surfactant in the mobile phase.

The mobile phase: 50% acetonitrile and aqueous buffer of pH 6.99 (acetic acid, phosphoric acid, and boric acid (2.74 mM each), and sodium hydroxide (8.60 mM)) and various concentration of SDS (range from 9.375 mM to 150 mM), 0.01% m/v solution of azorubine, determination at 510 nm, equipment: Spectrophotometer Genesis 20 VIS.
